# A tyrosine phosphatase SHP2 gain-of-function mutation enhances malignancy of breast carcinoma

**DOI:** 10.18632/oncotarget.6561

**Published:** 2015-12-10

**Authors:** Zhongqian Hu, Xinyi Wang, Haoshu Fang, Yakun Liu, Danlei Chen, Qian Zhang, Xia Liu, Daoyan Wei, Chengkui Qu, Siying Wang

**Affiliations:** ^1^ Department of Pathophysiology, Anhui Medical University, Hefei 230032, China; ^2^ Department of Ultrasound, Zhongda Hospital, Southeast University, Nanjing 210009, China; ^3^ Department of Clinical Medicine, Anhui Medical University, Hefei 230032, China; ^4^ Department of Pediatrics, Aflac Cancer and Blood Disorders Center, Emory University School of Medicine, Atlanta 30322, GA, USA

**Keywords:** breast cancer, SHP2, D61G mutation, tumor, gain-of-function

## Abstract

**Background:** Evidence suggests that Src homologous protein phosphotyrosyl phosphatase 2 (SHP2) mutations promote cancer development in several solid tumours. In this study, we focused on the *in vivo* and *in vitro* effects of an SHP2 mutation on the breast cancer phenotype to determine whether this mutation is correlated with a malignant phenotype.

**Methods:** Mutant PTPN11 cDNA (D61G) was transduced into MDA-MB231 and MCF-7 cells. The effects of the D61G mutation on tumourigenesis and malignant behaviours, such as cell adhesion, proliferation, migration and invasion, were examined. Potential underlying molecular mechanisms, i.e., activation of the Gab1-Ras-Erk axis, were also examined.

**Results:** *In vitro* experiments revealed that tumour adhesion, proliferation, migration and invasion were significantly increased in the SHP2 D61G mutant groups. Consistently, *in vivo* experiments also showed that the tumour sizes and weights were increased significantly in the SHP2 D61G-MB231 group (p < 0.001) in association with tumour metastasis. Mechanistically, the PTPN11 mutation resulted in activation of the Ras-ErK pathway. The binding between Gab1 and mutant SHP2 was significantly increased.

**Conclusion:** Mutant SHP2 significantly promotes tumour migration and invasion at least partially through activation of the Gab1-Ras-Erk axis. This finding could have direct implications for breast cancer therapy.

## INTRODUCTION

Breast cancer is the most common malignant tumour affecting women worldwide. It is the second leading cause of cancer-caused mortality in women in the US. The most important factors contributing to this high mortality rate are metastasis and recurrence. Studies have reported the overexpression of Src homologous protein phosphotyrosyl phosphatase 2 (SHP2SHP2) in breast cancer tissues. However, the role of the SHP2 signalling pathway in the malignant progression of breast cancer remains unknown.

The protein tyrosine phosphatase (PTP) SHP2 is encoded by the PTPN11 gene, which relays signals from growth factor receptors to Ras and other effectors [1, 2]. SHP2 is a non-membranous PTP with largely positive regulatory roles in the signal transduction of many growth factors, cytokines, and hormones. Dominant negative SHP2 has been reported to disrupt *Xenopus* gastrulation and to cause tail truncation [3, 4]. The targeted deletion of exon 3 of SHP2 has been shown to result in decreased cell spreading and migration [5, 6] and impaired limb development in chimeric mice [7]. Roles of SHP2 in cell adhesion and migration have also been demonstrated using catalytically inactive SHP2SHP2-overexpressing cells [8, 9]. However, the molecular mechanisms by which SHP2 promotes these cellular processes have not been well defined. For example, the role of SHP2 in activation of members of the Rho family of small GTPases, which are critical for cell motility, remains controversial. In this context, both positive [2, 10] and negative roles [11, 12] of SHP2 have been reported. This discrepancy may be due to differences in the cell models used in analyses. In cells over-expressing catalytically inactive SHP2SHP2, the catalytic activity of endogenous SHP2 is inhibited. However, as SHP2 also functions independent of its catalytic activity, the overexpression of catalytically deficient SHP2 may increase its scaffolding function [13, 14].

Gain-of-function (GOF) mutations in SHP2 lead to the dysregulation of multiple signalling pathways, thereby contributing to the development of different human disorders [15]. Studies have demonstrated that PTPN11 GOF mutations are sufficient to drive the development of juvenile myelomonocytic leukaemia (JMML)-like myeloproliferative disorder and malignant acute leukaemia in mice. Of note, most human SHP2SHP2 mutations occur in the N-SH2 or PTP domain and involve the deletion of residues that participate in basal inhibition. The most common SHP2 GOF mutations are D61G [16] and E76D, which are present in approximately 50% of patients with Noonan syndrome (NS), a developmental disorder associated with an elevated risk of JMML [17, 18]. The phenotypes resulting from loss of SHP2 function are attributed to the roles of SHP2 in cell signalling pathways induced by growth factors and cytokines [19, 20]. SHP2 generally promotes signal transmission during growth factor/cytokine signalling in both catalytic-dependent and catalytic-independent manners [21, 22]. A positive role of SHP2 in intracellular signalling processes, particularly the MAPK-PI3K kinase pathway, has been well established. However, the exact underlying mechanism remains elusive [23, 24]. In addition, somatic GOF mutations within the PTPN11 gene have been found to commonly occur in certain solid tumours, such as colon carcinoma, breast cancer, lung cancer, thyroid cancer, melanoma, and neuroblastoma [15, 25, 26]. However, the manner by which GOF mutations in SHP2 induce these phenotypes is not fully understood. In this study, we introduced a GOF mutation into the SHP2 in breast cancer cell lines, and these cell lines were used to investigate the roles of GOF mutations in SHP2 in the malignant behaviours of mammary tumours *in vitro* and *in vivo*. Our results provide novel insights into the potential role of SHP2 in breast oncogenesis and also suggest that the suppression of excessive SHP2 expression or activity may be a novel therapeutic strategy for breast cancer patients.

## RESULTS

### SHP2 protein expression in transfected breast cancer cells

**Figure 1:**
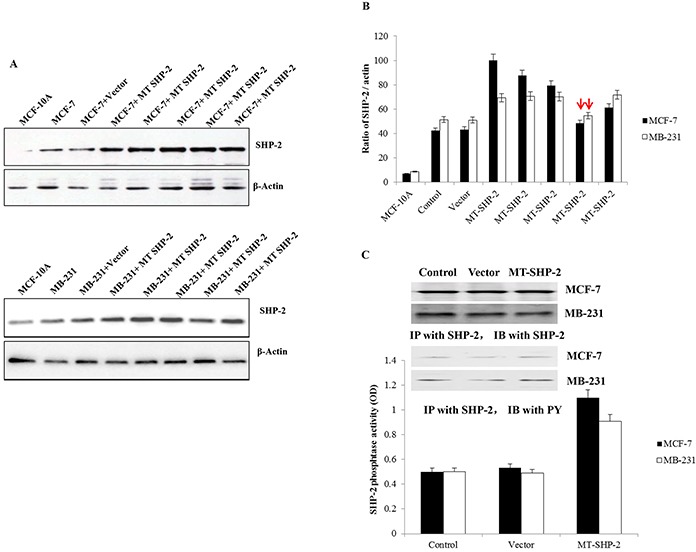
MDA-MB231 and MCF-7 cells transfected with pcDNA3.1SHP2D61G **A.** Western blot analysis was performed to examine SHP2 expression after transfection in MCF-7 cells (upper panel) and MB-231 cells (lower panel). **B.** The the bands were analysed by ImageJ. The expression ratio of SHP2 to actin is presented. The bars with red arrows indicate similar SHP2 expression levels compared with wild-type cells, and these cells were chosen for future experiments. **C.** Western blot and IP analyses were performed to confirm SHP2 expression in colonies, which were selected for further experiments. The data are presented as the mean ± SD.

We examined SHP2 expression in each of the transduced populations. After transfection with pcDNA3.1 or pcDNA3.1SHP2SHP2^D61G^, G418-resistant MDA-MB231 and MCF-7 clones were expanded in culture. Four weeks later, 6 colonies were selected to examine SHP2 expression by western blot analyses. As shown in Figure 1A, positive SHP2 expression was detected in the cells transduced with mutant SHP2 plasmid. Transfection was confirmed by the DNA sequencing of selected clones (Figure 1). The level of SHP2 protein was normalised to that of the constitutively expressed β-actin protein by densitometry (Figure 1B). To investigate the effect of mutant SHP2 on tumour development, the clones with similar SHP2 expression levels as the control group were chosen for further study. In addition, phosphatase activity was measured, and the results indicated that it was increased in the mutant groups compared with the control groups.

### PTPN11 mutation promotes cell adhesion and extension

**Figure 2:**
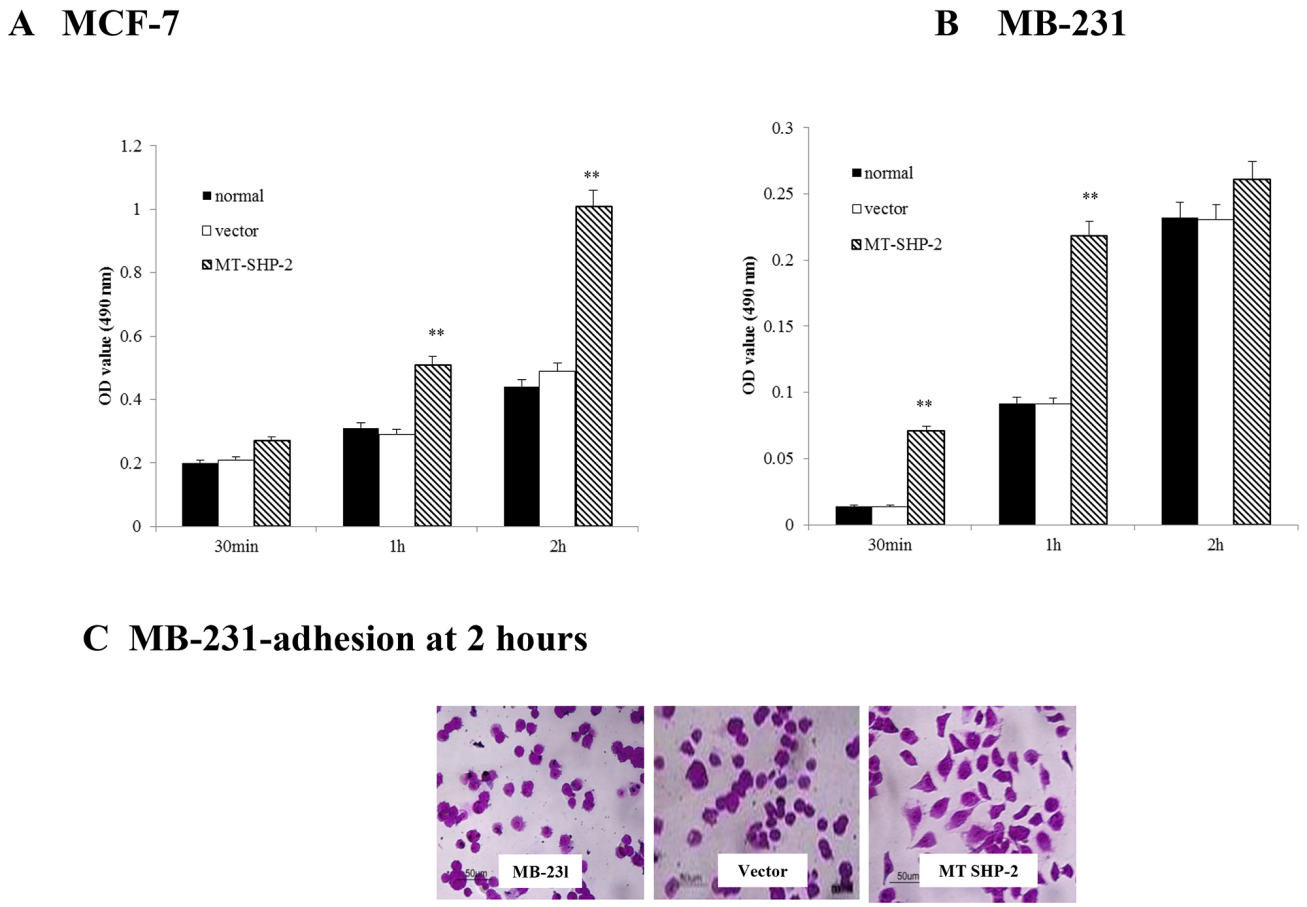
PTPN11 mutation enhances breast cancer cell adhesion to FN **A.** and **B.** FN-adhesive cells were detected by performing crystal violet staining; the absorbance values were compared at 490 nm to assess the adhesion of the different groups of MCF-7 (A) and MB-231 cells (B). **C.** Morphological evaluation of adhesive cells under high magnification, original magnification was 400×. The data are presented as the mean ± SD. Three independent experiments were performed. *p < 0.05.

We examined the abilities of SHP2SHP2-D61G-MB231 and MCF-7 cells to adhere to FN. Cells were seeded in 96-well plates previously coated with FN and incubated for 30 min, 60 min, or 2 h, respectively. After removal of non-adherent cells by washing with PBS, the attached cells were stained with crystal violet, and the absorbance of MCF-7 cells (Figure 2A) and MB-231 cells (Figure 2B) at 499 nm was measured. Transfection with the SHP2-D61G mutant significantly increased the adhesion of MCF-7 and MB-231 cells at 2 h. The amounts of adherent cells were similar in the non-transfected and vector-MB231 cell lines. However, the mutant cells appeared more elongated, whereas the non-transfected and vector-MB231 cells did not spread or elongate extensively and appeared smaller (cell morphology was only demonstrated for MB-231 cells; Figure 2C).

### PTPN11 mutation enhances migration, invasion and proliferation of breast cancer cells

**Figure 3:**
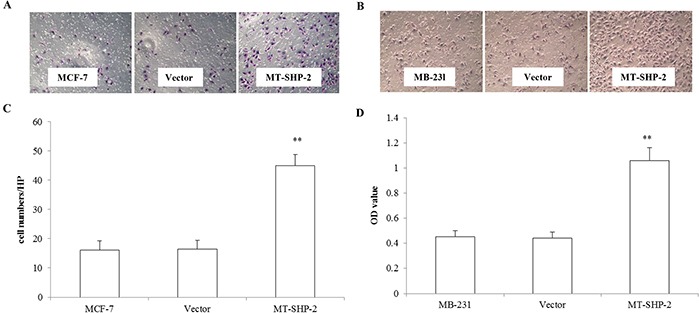
PTPN11 mutation enhances cell migration of MCF-7 cells and MDA-MB231 cell **A.** and **B.** Crystal violet staining of the membrane after Boyden chamber migration was presented of MCF-7 and MB-231 cells. **C.** and **D.** The absorbance values were compared at 490 nm to assess the migration of the different groups of MCF-7 and MB231 cells. The data are presented as the mean ± SD. Three independent experiments were performed. *p < 0.05.

**Figure 4:**
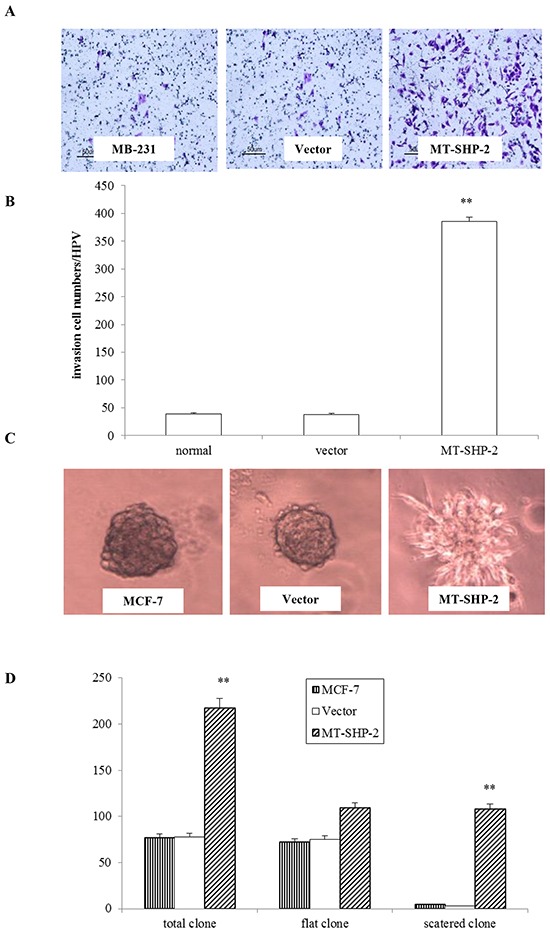
PTPN11 mutation enhances invasion of breast cancer cells **A.** In the transwell assay, invasiveness was quantified by assessing the migration of cells through the Matrigel into the bottom of the chamber. **B.** The total number of cells that crossed the membrane was counted. Positively stained cells were counted and analysed by randomly selecting 10 HPVs for each group. **C.** The Matrigel 3D culture experiment revealed the invasiveness of the MB231 cells that expressed the mutant SHP2. **D.** Statistical analysis of the 3D culture experiment results. The data are presented as the mean ± SD. All experiments were performed three times independently, *p < 0.05.

To comprehensively examine the effects of SHP2SHP2 on tumour development and progression, the migration of MB-231 and MCF-7 cells was assessed (Figure 3). Cells were seeded in the upper chamber of a transwell system and allowed to migrate into the lower chamber through the small pores of a membrane (8 mm). Cells that crossed the membrane were counted after 12 h for MB-231 cells and after 24 h for MCF-7 cells. The numbers of migrated cells in the mutant groups were significantly increased for both the MB-231 and MCF-7 cells compared with the control and vector transfected cells (Figure 3A and 3B; p < 0.005). To further investigate the effects of SHP2-D61G on cell invasion, we performed Matrigel adhesion assay to measure cells that passed through the Matrigel membrane barrier. As shown in Figure 4A and 4B, invasion of SHP2-D61G-MB231 cells was enhanced significantly compared with vector treated cells (p < 0.05), and 3D Matrigel assay revealed a similar trend (Figure 4C and 4D).

To further characterise the effects of mutant SHP2SHP2 on MDA-MB231 cells, additional studies were performed to examine its role in tumourigenesis. The *in vitro* assays included the measurement of foci formation, which reflects an increase in density-dependent growth, or the measurement of anchorage-independent growth in soft agar, as previously described. Both SHP2 MB-231 and SHP2 MCF-7 cells exhibited significantly enhanced foci formation, as multiple foci formed in the mutant SHP2SHP2 groups compared with the control groups (Supplementary Figure S2A and S2B). Anchorage-independent growth assays examining colony growth in soft agar revealed the presence of an increased number of colonies of SHP2-D61G-MB231 and MCF-7 cells compared with those of control cells (Supplementary Figure S2C-S2E).

The increased colony number and colony size observed in the colony formation assay provided additional evidence to support the notion that the SHP2 D61G mutation results in higher rates of self-renewal and proliferation compared with vector transfected cells, resulting in a high rate of tumourigenesis.

### The SHP2SHP2 GOF mutation promotes mammary tumour growth and metastasis in mice

**Figure 5:**
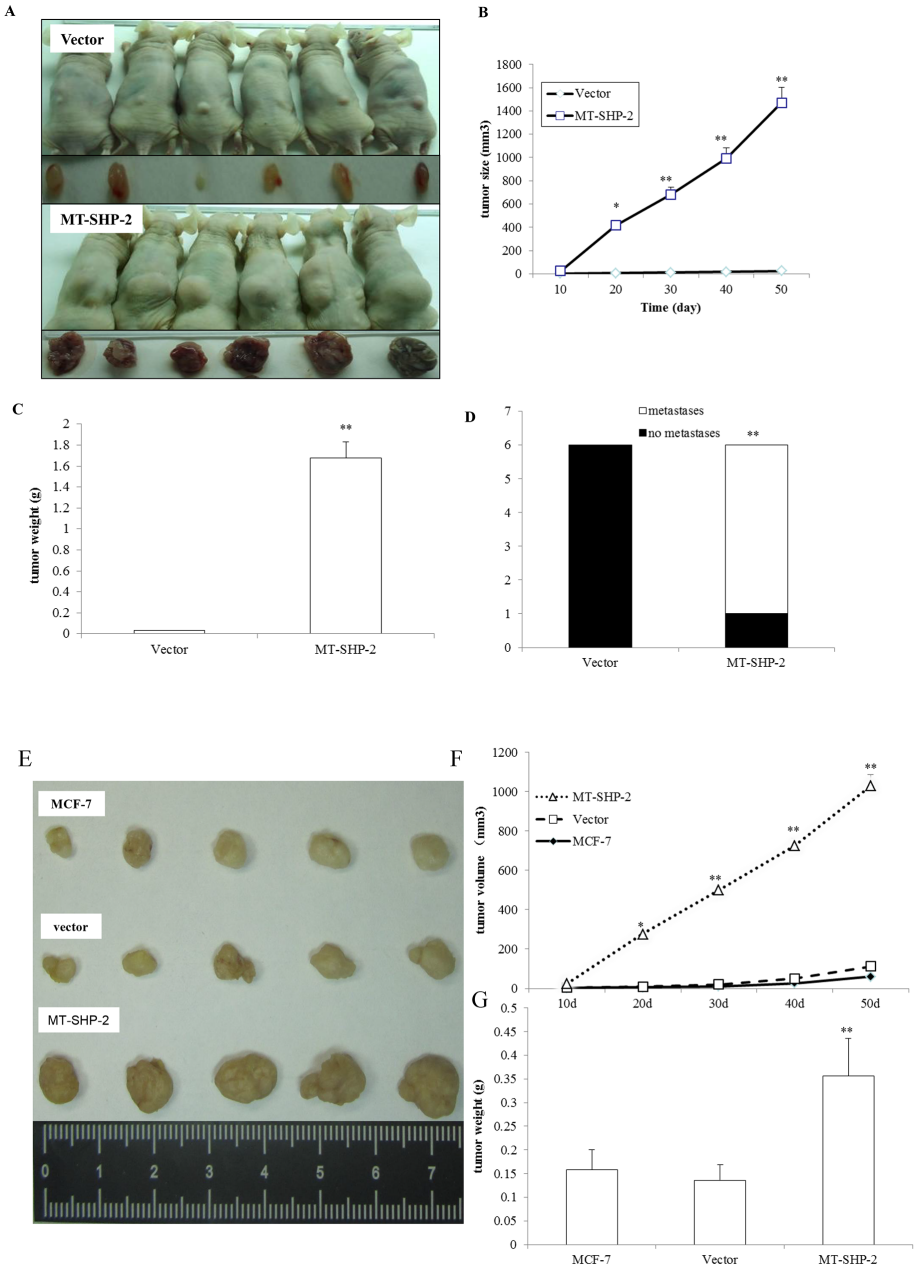
PTPN11 mutation promotes mammary tumour growth **A.** A representative image showing mammary tumours from the vector (upper panel) and SHP2 mutant (lower panel) groups for MB231 cells. Images were captured at the time point of sacrifice. **B.** Tumour size was monitored every 10 days after implantation for MB-231 cells, and it was calculated as indicated in the Materials and Methods section. **C.** Solid tumours from MB-231 cells were removed, and their weights were determined after sacrifice. **D.** Tumour metastasis to distant organs was assessed. **E.** Representative image showing mammary tumours from the vector (upper panel) and SHP2 mutant (lower panel) groups for MCF-7 cells. Images were captured at the time point of sacrifice. **F.** Tumour size was monitored every 10 days after implantation for MCF-7 cells. **G.** Solid tumours from MB-231 cells were removed, and their weights were determined after sacrifice. The data are presented as the mean ± SEM (n = 6/group) *p < 0.05.

To further confirm the observed *in vitro* effects of the GOF SHP2SHP2 mutation on the proliferation, viability, and invasiveness of human breast cancer cells, we investigated its roles in the growth and metastasis of mammary tumours *in vivo* using a tumour xenograft model. Human mammary adenocarcinoma cells were injected into BALB/c nude mice. Mammary tumours were detected approximately 1 week after initial implantation (Figure 5). The SHP2 GOF mutation enhanced tumour growth in the MB231 (Figure 5A-5D) and MCF-7 groups (Figure 5E-5G). As shown in Figure 5A and 5E, the growth rate of the mammary tumours from the SHP2 MB231 cells was significantly higher than that of the tumours from the control cells. The mammary tumours were removed after 50 days for further measurement. As illustrated in Figure 5B and 5C, the SHP2 GOF mutation significantly increased the weights of the mammary tumours. Similar results were observed using MCF-7 cells.

We further determined whether the SHP2 GOF mutation promotes mammary tumour metastasis. Internal organ metastasis was evaluated by histological analysis. As shown in Figures 5D and S3 and Table 1, kidney, liver, and lung metastatic carcinoma nodes were detected in all 6 mice in the SHP2 D61G-MB231 group, while metastases were not identified in any animals in the vector-MB231 group.

**Table 1:** Effects of SHP2 GOF mutation on tumour metastases in different organs of tumour xenograft model mice

Group/organ	Liver	Kidney	Lung	Aversion Rate
Vector	0	0	0	0
				Liver: 18%
MT-SHP2	1	3	2	Kidney: 50%
				Lung: 33%

### PTPN11 mutation results in constitutively elevated Ras-Erk signalling pathway activity and increased Gab1 binding

**Figure 6:**
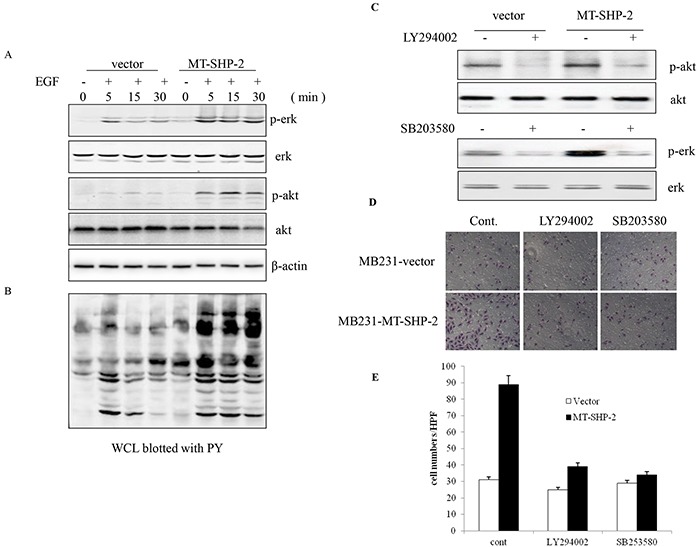
PTPN11 mutation increases Ras-Erk signalling pathway activation Cells were starved overnight and stimulated in DMEM with 10% FBS for 2 h. EGF stimulation induced SHP2 tyrosine phosphorylation. The cells were incubated for 5 min and 15 min in the presence or absence of EGF (5 ng/mL). **A.** Erk and AKT activation was detected by western blotting. **B.** Phosphotyrosine (PY) was detected in whole-cell lysates (WCLs). **C.** Western blotting was performed to detect the activation of p-AKT and p-ERK after treatment with LY294002 and SB203580. (D and E) FN-adhesive MCF-7 cells were visualised **D.** and counted **E.** after treatment with LY294002 and SB203580. Positively stained cells were counted and analysed by randomly selecting 10 HPVs for each group. The data are presented as the mean ± SEM (n = 6/group) *p < 0.05.

Many studies have shown that SHP2 is required to transduce signals to the downstream MEK/ERK and PI3K/AKT pathways to transform cells [23, 24, 27]. Therefore, we examined activation of the MEK/ERK and PI3K/AKT-dependent kinases following mutant SHP2 activation. Western blot analysis was performed to examine the levels of phosphorylated Erk and AKT. The SHP2 GOF mutation in MDA-MB231 cells resulted in a fast and dramatic increase in the levels of signalling molecules, including p-Erk and p-AKT (Figure 6). Conversely, the MAPK and PI3K inhibitors LY294002 and SB203580 blocked the activation of p-AKT and p-ERK (Figure 6C), leading to the decreased adhesion ability of MB231 cells in the SHP2 D61G group (Figure 6D and 6E).

**Figure 7:**
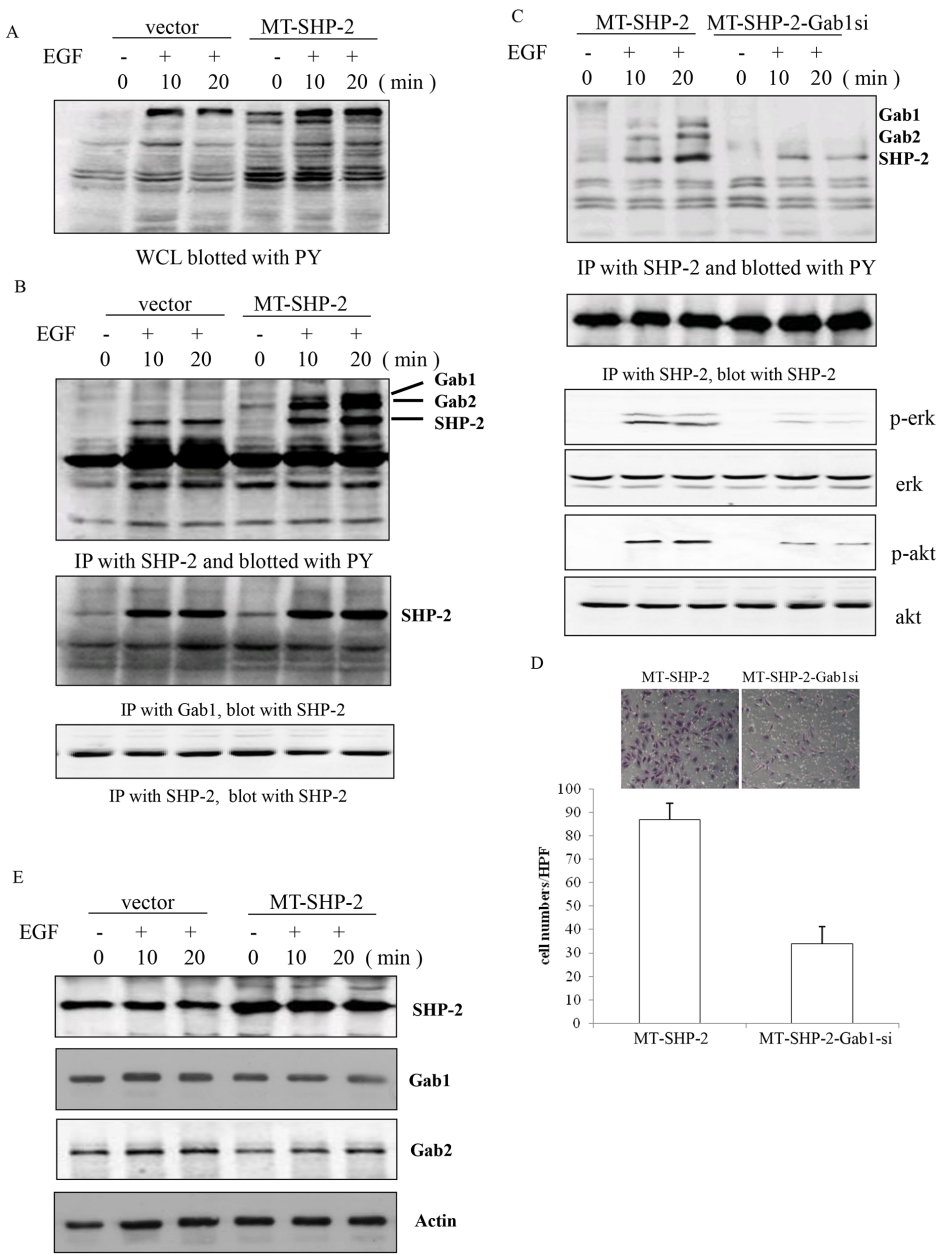
PTPN11 mutation increases the binding between Gab1 and SHP2 in MB231 cells **A.** PY activation was detected in WCLs from SHP2 mutant cells. **B.** Immunoprecipitation assay was performed to assess the interaction between Gab1 and SHP2. **C.** The activation of p-Erk and p-Akt was detected by western blotting after the silencing of Gab1 expression. **D.** The adhesion of MB-231 cells was detected after the silencing of Gab1 expression using siRNA. **E.** SHP2, Gab1, and Gab2 were identified using a specific antibody after EGF treatment. Positively stained cells were counted and analysed by randomly selecting 10 HPVs for each group. The data are presented as the mean ± SEM (n = 6/group) *p < 0.05.

Earlier studies have indicated that the enhanced activation of ERK and Akt is correlated with increased interaction between mutant SHP2 (SHP2 D61G) and the scaffolding protein Gab1. Conversely, a Gab1 mutant that is unable to bind SHP2 has been reported that does not potentiate MAPK activity [28]. Thus, we aimed to determine whether a GOF mutation in SHP2 potentiates the activation of p-AKT and p-ERK by altering the interaction of Gab1 and SHP2 in our system. Breast cancer cells were serum deprived for 24 h, followed by stimulation with EGF for varying amounts of time. Interestingly, we found that the binding between Gab1 and SHP2 was constitutively elevated compared with that between Gab2 and an empty vector in MB-231 cells (Figure 7A-7C, and 7E) and MCF-7 cells (Supplementary Figure S4). Gab1 siRNA was used to confirm this finding, and we found that the treatment of cells with Gab1 siRNA significantly decreased the binding between Gab1 and SHP2, resulting in down-regulation of the activation of p-Erk, and p-AKT (Figure 7C). More interestingly, de-activation of the Ras-Erk pathway led to the decreased adhesion ability of MB231 cells (Figure 7D).

Taken together, these data suggest that the SHP2 mutation promotes the increased binding between SHP2 and Gab1, thereby increasing activation of the Ras-Erk pathway.

## DISCUSSION

The results of the present study demonstrated that the D61G mutant, a known GOF mutant of SHP2, enhanced the interaction between Gab1 and SHP2 and increased activation of the MAPK-PI3K pathway, which in turn promoted tumour migration and invasion and other malignant behaviours.

Somatic SHP2 mutations are the major causes of JMML, NS, and several solid tumours [15, 25, 26]. Previous studies have suggested that SHP2 GOF mutations are important in the pathogenesis of these diseases. However, the biochemical functions of these mutants are not fully understood.

We have previously characterised the mechanisms by which SHP2 overexpression promotes cell adhesion, spreading, and movement in breast cancer [29]. The results of the present study demonstrated that the up-regulation of SHP2 promoted binding between Gab2 and SHP2, in addition to cell proliferation. Further, the MDA-MB231 and MCF-7 cells transduced with SHP2 D61G displayed enhanced adhesion/spreading. In addition, the SHP2 GOF mutation enhanced proliferation, as demonstrated by colony formation and anchorage-independent colony formation assays. These observations suggest that the heterozygous GOF mutations in SHP2 that have been identified in human diseases sufficiently enhance cell growth in a dominant manner by these cellular processes.

The effects of SHP2 GOF mutations on breast cancer cells might occur though the Gab1-ERK signalling axis. Previous studies have indicated that the enhanced activation of ERK and Akt is correlated with increased interaction between mutant SHP2 (SHP2 D61G) and the scaffolding protein Gab1 [23, 24, 27]. This finding was further confirmed by decreasing Gab1 expression, which resulted in inhibition of MAPL/PI3K signalling pathway activation. The increased interaction between mutant SHP2 and Gab1 is likely due to the conformational change of SHP2 induced by the D61G mutation. The D61G mutation disrupts the intramolecular binding between the N-terminal SH2 domain (N-SH2) and the PTP domain, which leads to high-affinity binding between SHP2 and Gab1 because there is no free-energy cost associated with disruption of the N-SH2/PTP interface and the opening of the N-SH2 phosphotyrosine binding pocket [30–33]. Similarly, in SHP2 D61G-MB231 cells, the EGF-induced ERK and PI3K/Akt pathway activities were markedly enhanced. This result supports the generally positive effects of SHP2 catalytic activity on ERK and Akt signalling. However, the underlying mechanisms remain to be further elucidated.

Using a mammary tumour xenograft model, we also demonstrated that the SHP2 GOF mutation promoted tumour growth and metastasis in nude mice. Tumour weights and sizes were significantly increased in the SHP2 mutant groups compared with the control groups. Increases in lung, liver and kidney metastases were also observed. These observations indicate that the SHP2 mutation promotes tumour growth and metastasis *in vivo*.

In conclusion, our results have revealed that the SHP2 GOF mutation promotes tumour migration and invasion. This effect of SHP2 might occur via an increase in the interaction between Gab1 and SHP2, thereby promoting activation of the MAPK-PI3K signalling pathway. These findings clearly establish a role of the SHP2 GOF mutation in breast tumour growth and provide novel insights into the mechanisms of SHP2 GOF mutant-mediated tumour promotion. Further research should focus on identification of a therapeutic target in the Gab1-ERK signalling pathways to inhibit the development of breast cancer and exploration of novel preventative and treatment strategies for this disease.

## MATERIALS AND METHODS

### Cell culture and transfection

MDA-MB231 and MCF-7 human breast cancer cells were cultured in the Dulbecco Eagle's minimum essential medium (DMEM) supplemented with 10% heat-inactivated foetal bovine serum (FBS). MDA-MB231 and MCF-7 cells were transfected with an empty pcDNA3.1 vector or a SHP2 D61G-pcDNA3.1 vector using Lipofectamine 2000 reagent (Invitrogen, Carlsbad, US). After 24 h, the medium was replaced with fresh DMEM containing 800 μg/μl G418. The culture medium was replaced three times a week until stable transfected colonies emerged. Multiple clones were pooled and expanded in culture. All experiments were repeated with at least two independent transfectant pools for each construct. This study was conducted with approval of the institutional review board of Anhui Medical University.

### Phosphatase activity analysis

Phosphatase activity was measured with an alkaline phosphatise yellow (pNPP) substrate system using a commercial ELISA kit (Sigma-Aldrich, St. Louis, US). All procedures were performed according to the manufacturer's instructions. ELISA measurements were performed in 96-well polystyrene plates using an ELISA plate reader (Thermo Scientific, Waltham, US) at 405 nm.

### Preparation of cell lysates

Cells were stimulated with 5 ng/ml EGF for a defined time period. Whole-cell lysates were obtained by scraping cells into hypotonic lysis buffer (Sigma-Aldrich, St. Louis, US), followed by centrifugation at 13000 g for 15 min at 4°C. The protein concentration was determined by BCA assay.

### Immunoprecipitation analysis

A cross-link immunoprecipitation kit was purchased from Thermo Pierce. Immunoprecipitation experiments were performed according to the manufacturer's instructions. Briefly, an SHP2 antibody (10 μg, Cell Signaling Technology, Beverly, US) was first incubated with protein A/G plus agarose resin (20 μl), and it was then covalently cross-linked onto the resin by incubation with 450 μM DSS at room temperature for 1 h. Effluent obtained after 24 h of cold storage was first precleared using control agarose resin to reduce nonspecific protein binding. Precleared effluent was incubated with protein A/G beads coupled to anti-SHP2 (Cell Signaling Technology, Beverly, US) at 4°C overnight. The effluent was eluted from the beads using antigen elution buffer for Western blot analysis.

### Western blot analysis

Western blot analysis was performed as previously described [34]. Briefly, 15 μg of the protein samples were separated by electrophoresis on an SDS-polyacrylamide gel. The separated proteins were transferred onto nitrocellulose membranes. Then, the membranes were blocked in PBST (Invitrogen, Carlsbad, US) at room temperature for 1 h. The membranes were subsequently probed overnight at 4°C with the following primary antibodies directed against the target proteins: rabbit anti-SHP2 antibody (1:1000, Cell Signaling Technology, Beverly, US), rabbit anti-Gab1 antibody (1:500, Abcam, Cambridge, UK), p-ERK (1:1000, Cell SignCell Signaling Technology, Beverly, US), ERK (1:1000, Cell Signaling Technology, Beverly, US), p-AKT (1:1000, Cell Signaling Technology, Beverly, US), AKT (1:1000, Cell Signaling Technology, Beverly, US), and β-actin (1:5000, Abcam, Cambridge, UK). After three washes in PBST, the membranes were incubated with secondary antibodies conjugated to horseradish peroxidase (GE-healthcare, Waukesha, US). Signals were detected using a chemiluminescence method (GE-healthcare, Waukesha, US). The membranes were then stripped with stripping buffer for 15 min at room temperature and immunoblotted with an anti-actin antibody. Film digitalisation was performed using ImageJ 1.43 G software (NIH, Bethesda, MD, USA).

### Cell adhesion assay

Cancer cells were collected after trypsinisation and washed in serum-free DMEM containing 0.2% trypsin inhibitor. The cells were re-suspended at a concentration of 1×10^5^ cells/ml in DMEM, and 100 μl of cell suspension was added to each well of 96-well plates that had been coated overnight at 4°C with 10 mg/ml fibronectin (FN) and blocked with 1 mg/ml bovine serum albumin. After 30 min, 1 h, or 2 h, non-adherent cells were removed by washing with phosphate-buffered serum (PBS), and attached cells were then stained with 0.1% crystal violet in 20% methanol. After washing with PBS, the crystal violet stain was eluted with 0.1 M sodium citrate, and optical absorbance was measured at 499 nm using a microplate reader.

### Cell migration and invasion assays

Cell migration was analysed using a Transwell System (Costar, NY, US) as previously described [45]. Transwell chambers with polycarbonate filters of 8-μm porosity (BD Biosciences) were used in this study. In invasion assay, the chambers were pre-coated with 100 μl of 5 mg/ml Matrigel (BD Biosciences, Franklin Lakes, US) and loaded into 24-well culture plates. In migration assay, the chambers were loaded into 24-well culture plates without Matrigel pre-coating. The transwell insert consisted of upper and lower chambers separated by a membrane with an 8-μm pore size. MDA-MB231 cells (1×10^5^) were trypsinised and plated in the upper chamber with DMEM containing 2% FBS. The lower chamber was filled with medium containing 15% FBS. The chambers were then incubated at 37°C with 5% CO_2_ for 12 h. The membrane was fixed with methanol, and the cells remaining on the upper chamber were removed. The migrated cells were stained with Giemsa and counted. For each well, five random microscopic fields were counted. Data were obtained from triplicate wells for analysis.

### Cell growth assay

Anchorage-independent growth, which is a characteristic of *in vitro* tumourigenicity, was assessed using soft agar clonogenic assays. Cells were detached and plated in 0.6% agarose (1×10^3^ cells/well in 6-well plates) with a 1.2% agarose underlay. The number of colonies was counted after 18 days. For focus formation assay, cells were reseeded and cultured for 4 weeks in DMEM with 10% FBS. The cells were then fixed with formalin and stained with 0.1% crystal violet.

### Three-dimensional morphogenesis assay

To investigate the effect of tumour angiogenesis, we utilised a three-dimensional (3D) model. Eight-well chamber slides were pre-coated with 150 μl Matrigel. A total of 2.5×10^3^ mutant or control MCF-7 cells were re-suspended in DMEM medium and were then seeded on top of the coated chamber slides. The cells were cultured for 30 min, and then the media was replaced with 150 μl of 5% Matrigel. After 15 min of incubation, 500 μl DMEM was added. The top layer of mediumwas changed every four days.

### *In vivo* tumourigenicity assay

Tumour cells (2×10^6^) were diluted with PBS to a total volume of 0.1 ml and injected into the mid-dorsa of BALB/c nude mice (4-6 weeks old). The animals were inspected weekly for tumour development. Growing tumours were measured using Vernier callipers, and tumour volumes were calculated using the formula volume = length × width^2^ × 10. Based on the tumour volume data, the mice were sacrificed, and the tumours were isolated at 50 days post-injection. Upon tumour removal, tumour volumes were calculated using the equation tumour volume = (length × width^2^)/2. The tumour samples were fixed in 10% neutral buffered formalin for further analysis.

### Histological staining

Immunohistochemical procedures were performed as previously described [46]. Tumour samples were fixed in 10% neutral buffered formalin for at least 24 h. Paraffin embedding was performed, and the sections (3 μm) were cut and stained with haematoxylin and eosin (H&E).

### Statistical analyses

All experiments were repeated a minimum of 3 times. The data are shown as the mean value ± standard deviation. The groups were compared using one-way ANOVA, followed by Pearson's coefficient analysis of bivariate correlation. A p-value of below 0.05 was considered significant.

## SUPPLEMENTARY FIGURES


