# Supplementary material for: A tyrosine phosphatase SHP2 gain-of-function mutation enhances malignancy of breast carcinoma
Source: Oncotarget. 2015 Dec 10;7(5):5664–76. doi: 10.18632/oncotarget.6561 (PMC4868712; doi:10.18632/oncotarget.6561)
Supplement: Supplementary file 1 [file oncotarget-07-5664-s001.pdf]

SUPPLEMENTARY FIGURES

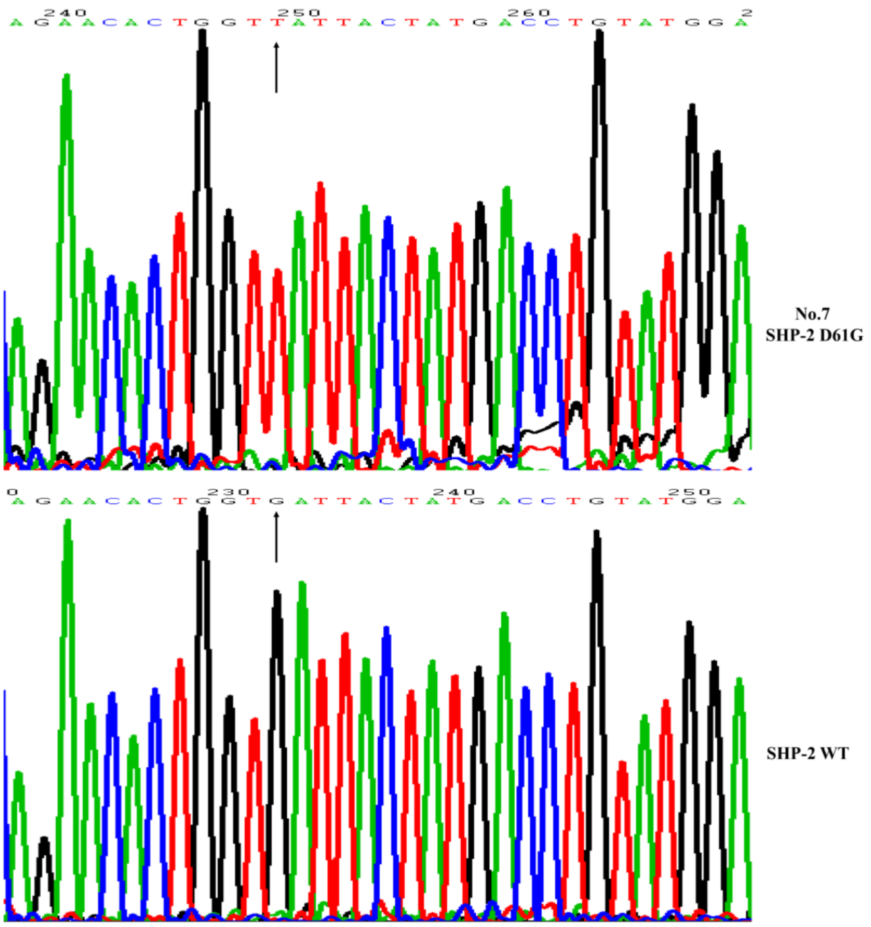

Supplementary Figure S1: Sequencing of SHP2 expression in MB-23 cell line. Transfection was confirmed by the DNA sequencing of selected clones (upper panel) and a comparison of their sequences with that of SHP2 in the wild-type control (lower panel).

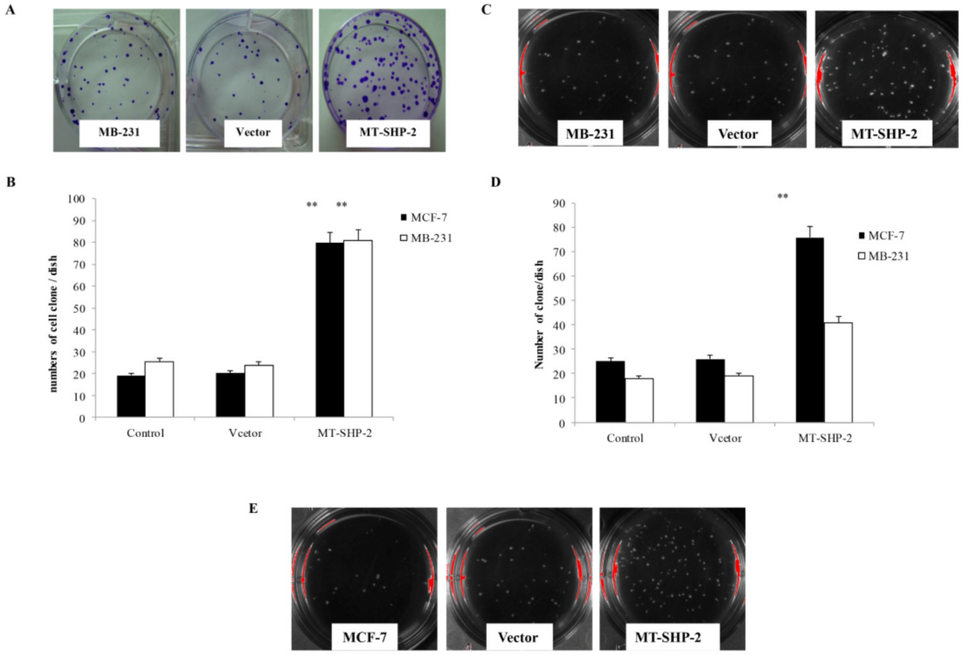

Supplementary Figure S2: PTPN11 mutation enhances colony formation and growth in soft agar of breast cancer cells. A. Colony formation assay of normal, vector control, and SHP2 D61G mutant MB231 cells. **B.** Positive clone counts for MB-231 and MCF-7 cells, respectively. **C.** and **E.** Anchorage-independent growth of MB-231 (C) and MCF-7 cells in soft agar was measured by colony growth assay (E). (C) The counting of clones of both MCF-7 and MB-231 **D.** cells. The data are presented as the mean ± SD of triplicate samples. All experiments were performed three times independently, $*p < 0.05$.

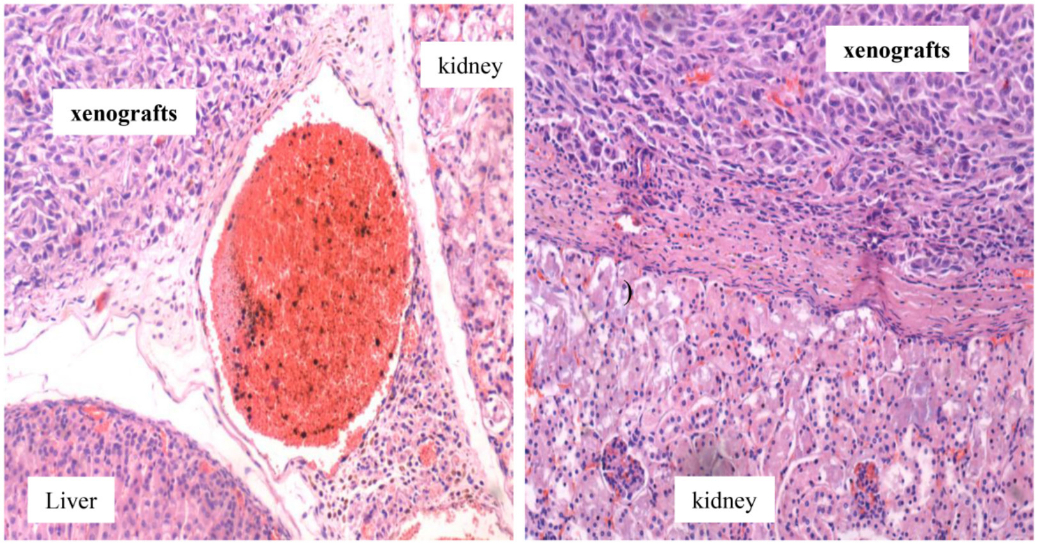

Supplementary Figure S3: Histological evaluation of the metastasis of breast tumours formed in abdominal cavity. The microphotograph shows metastatic carcinoma nodes in the kidneys, livers, and abdominal cavities of mice implanted with SHP2 D61G-MB231 cells.

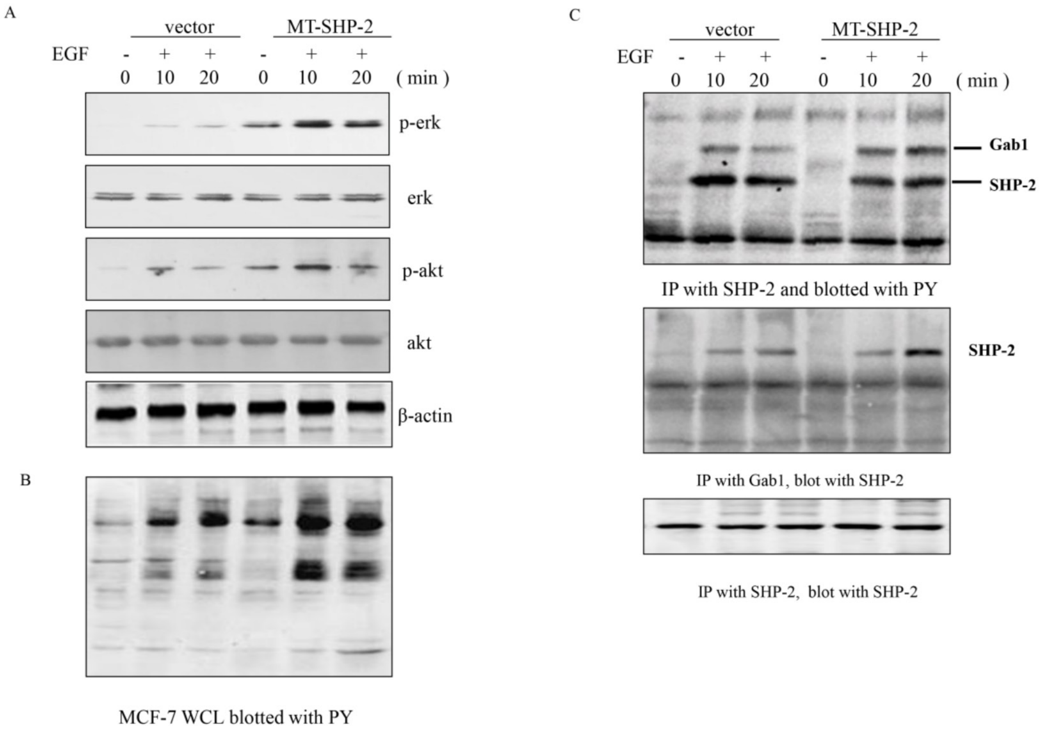

Supplementary Figure S4: PTPN11 mutation increases the binding between Gab1 and SHP2 in MCF-7 cells. A. The activation of p-Erk and p-Akt was compared between normal and mutant MCF-7 cells by western blotting. PY activation was detected using WCLs from SHP2 mutant cells. **B.** and **C.** Immunoprecipitation assay was performed to assess the interaction between Gab1 and SHP2. The data are presented as the mean ± SEM (n = 6/group) *p < 0.05.
